# An Overview of Normally-Off GaN-Based High Electron Mobility Transistors

**DOI:** 10.3390/ma12101599

**Published:** 2019-05-15

**Authors:** Fabrizio Roccaforte, Giuseppe Greco, Patrick Fiorenza, Ferdinando Iucolano

**Affiliations:** 1 Consiglio Nazionale delle Ricerche—Istituto per la Microelettronica e Microsistemi (CNR-IMM) Strada VIII, n. 5-Zona Industriale, Catania 95121, Italy; giuseppe.greco@imm.cnr.it (G.G.); patrick.fiorenza@imm.cnr.it (P.F.); 2 STMicroelectronics, Stradale Primosole 50, Catania 95121, Italy; ferdinando.iucolano@st.com

**Keywords:** gallium nitride, normally-off HEMT, power electronics

## Abstract

Today, the introduction of wide band gap (WBG) semiconductors in power electronics has become mandatory to improve the energy efficiency of devices and modules and to reduce the overall electric power consumption in the world. Due to its excellent properties, gallium nitride (GaN) and related alloys (e.g., Al_x_Ga_1−x_N) are promising semiconductors for the next generation of high-power and high-frequency devices. However, there are still several technological concerns hindering the complete exploitation of these materials. As an example, high electron mobility transistors (HEMTs) based on AlGaN/GaN heterostructures are inherently normally-on devices. However, normally-off operation is often desired in many power electronics applications. This review paper will give a brief overview on some scientific and technological aspects related to the current normally-off GaN HEMTs technology. A special focus will be put on the p-GaN gate and on the recessed gate hybrid metal insulator semiconductor high electron mobility transistor (MISHEMT), discussing the role of the metal on the p-GaN gate and of the insulator in the recessed MISHEMT region. Finally, the advantages and disadvantages in the processing and performances of the most common technological solutions for normally-off GaN transistors will be summarized.

## 1. Introduction

Nowadays, one of the most important societal challenges is represented by the steady increase of the energy consumption in the world. In fact, the energy consumption is expected to increase by about 40% worldwide in the next 20 years [1], when the electricity will cover the largest fraction (up to 60%) of the used energy. In this context, power electronics is the technology devoted to the control and management of electric power. In fact, power electronics systems are used to provide the optimal characteristics of electric power (i.e., current, voltage, frequency, etc.) for any targeted application. 

For more than five decades, silicon (Si) has been the dominant semiconductor for power electronics devices. However, today the continuous demand for higher current, voltage and power density capability, as well as the need of a better energy efficiency to reduce the global energy consumption, are the driving forces to introduce new semiconductor technologies in power electronics and to overcome the inherent limitations of Si-based devices. 

Wide band gap (WBG) semiconductors, like silicon carbide (4H-SiC) and gallium nitride (GaN), are considered the best materials for the future energy efficient power electronics [2]. However, while 4H-SiC [3] is mature in terms of crystalline quality and available device performances, gallium nitride (GaN) is still affected by several materials and technology concerns, limiting its full exploitation in power electronics applications [4].

This review paper will give a brief overview on some scientific and technological issues associated with the fabrication and performances of normally-off GaN-based high electron mobility transistors (HEMTs). In particular, the emphasis will be put on the p-GaN gate and on the recessed gate hybrid metal insulator semiconductor high electron mobility transistor (MISHEMT) approaches, which are the most widely adopted technologies at either R&D or commercial level. 

## 2. Gallium Nitride: Properties, Power Electronics Applications and Potential Market 

Gallium nitride (GaN) is a wide band gap semiconductor whose stable crystalline structure is the hexagonal wurtzite. Table 1 reports the relevant physical and electronic properties of GaN, compared with those of silicon (Si) and silicon carbide (4H-SiC). As can be seen, the wide band gap (3.4 eV) results in an intrinsic carrier concentration *n_i_* that is several orders of magnitude lower than in Si. Consequently, GaN devices should have a lower leakage current and give the possibility to operate at higher temperatures. Other key aspects are the high critical electric field and the maximum reachable breakdown of the material, making GaN a promising semiconductor for high voltage devices. In particular, the possibility to obtain a targeted breakdown voltage (*V_B_*) with thinner drift layers results in a significant reduction of the specific on-resistance (*R_ON_*) with respect to Si devices. In this way, devices that are more compact can be fabricated, minimizing both the static and dynamic losses. Finally, GaN devices are expected to have a high-frequency switching capability, due to the high saturation electron velocity of the material. High frequency operation is possible also due to the presence of the two-dimensional electron gas (2DEG) in AlGaN/GaN heterostructures [5], whose mobility values typically exceed 1000 cm^2^·V^−1^·s^−1^. Table 1 reports also some figures of merit (FOM) used to quantify the performance of high power and high frequency devices, normalized with respect to silicon: Johnson’s (JFOM), Baliga FOM (BFOM) and Baliga high frequency FOM (BHFOM) [6]. They quantify the maximum capability to energize carriers by electric field (JFOM), the minimum conduction losses during DC operation (BFOM), and the minimum conduction losses during high-frequency operation (BHFOM) [6]. The high values of the FOM clearly indicate the potential advantages offered from GaN devices with respect to the corresponding Si devices in high power and high frequency applications.

High electron mobility transistors (HEMTs) are lateral devices whose working principle is based on the presence of the 2DEG [9]. In the presence of the 2DEG acting as conduction channel, these devices are naturally normally-on, i.e., a current will flow between the source and drain electrodes even at zero bias to the gate (V_g_ = 0). Hence, the current is modulated by the application of a negative bias to a Schottky gate electrode. 

GaN-based HEMTs can be employed as switching devices in a large variety of high power applications. However, as will be specified in Section 3, normally-off transistors are preferred to guarantee a safe operation in power electronics systems.

**Figure 1:**
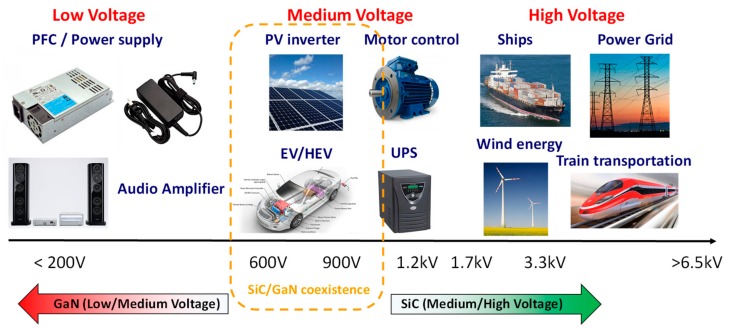
Possible applications of GaN power devices as a function of the voltage. The range of application of silicon carbide (SiC) devices is also shown for comparison.

Figure 1 schematically illustrates the potential applications of GaN devices in power electronics in the low-, medium- and high-voltage range. For comparison, the typical range of application of the other wide band gap semiconductor SiC is also indicated. 

As recently reported by several market analysts, GaN is better suited for the low-medium voltage range (200–600 V), which includes a large portion of the consumer electronic market (e.g., computer power supplies, audio amplifiers, etc.). Indeed, in this voltage range, the material is indicated as the best candidate to replace the existing Si devices. Clearly, the 600–900 V voltage range is strategic, as it covers the converters for electric (EV) and hybrid electric vehicles (HEV), as well as inverters for renewable energy (e.g., photovoltaic). In this voltage range, GaN devices are expected to be in competition or to coexist with SiC ones. Finally, for the high voltage applications (> 1.2 kV), e.g., industrial applications, trains/ships transportation, electric energy distribution grids, etc.), 4H-SiC remains at the moment the preferable choice, owing to a better material quality and device reliability. The future applications of GaN for high-voltage devices will strongly depend on the improvement of the material quality and the development of vertical devices based on bulk GaN [10]. 

In this wide scenario, SiC and GaN are expected to grow in share in the future of power electronics, as each material will enter in different applications, penetrating the market with a different speed. However, in power electronics, GaN is still a path away from SiC. In fact, the material is less mature and, hence, it is currently less adopted. For that reason, the inclusion of GaN devices in power electronics products must be still justified by high benefits. In this context, the reliability is presently a major challenge for the application of GaN in power systems. 

Despite all the current physical and technological issues related to GaN, according to recent reports of market analysts [11], a bright future is envisaged for GaN power devices market, which is expected to exceed $450 M in 2022. In this context, normally-off GaN-based HEMTs on large area Si substrates will play the dominant role, as the diffusion of vertical GaN power devices in the medium term will be still limited by the prohibitive cost of high quality large area bulk substrates. 

## 3. Normally-Off GaN HEMT Technology

As introduced in Section 2, the normally-on GaN HEMT operation (i.e., the achievement of transistors with a negative threshold voltage, V_th_ < 0) lies in the nature of an AlGaN/GaN heterostructure, due to the presence of the 2DEG at the interface. However, normally-off switching devices are preferred in power electronics, as they offer more failsafe operation conditions and gate-driver circuitry simplicity [12,13,14]. For this reason, both the academic community and many industrial players (e.g., Panasonic, Infineon, GaNSystems, OnSemiconductors, STMicroelectronics) are currently addressing their research efforts on the development and commercialization of reliable normally-off technologies. 

Under the physical point of view, to induce a positive shift the threshold voltage V_th_ and, hence, to obtain the desired normally-off HEMT behaviour, the region near the gate must be appropriately modified, e.g., by near-surface processing, or band engineering techniques. 

The first solution proposed to achieve a normally-off HEMT was the “recessed gate” approach [15], which consists in the local reduction of the AlGaN barrier layer thickness under the gate. Below a certain AlGaN thickness, the Fermi level at the interface will lie below the AlGaN conduction band minimum, thus corresponding to the depletion of the 2DEG and to a positive threshold voltage V_th_ [15,16]. 

Another early approach has been the “fluorine gate” HEMT [17], consisting in the introduction of negatively charged fluorine ions below the gate electrode, either by plasma or ion-implantation processes. In fact, the introduced negative charge produces a positive shift of the V_th_ and a depletion of the 2DEG [18]. Furthermore, the negative fixed charges (i.e., the incorporated F-ions) will lead to an upward conduction band bending of the AlGaN, causing an increase of the metal/AlGaN barrier height and, in principle, a reduction of the gate leakage current [12]. 

A further evolution of these approaches has been to combine the recessed gate layout with the introduction of fluorine below the gate [19]. In this case, a dielectric layer is also introduced below the metal gate in the recessed region to reduce the leakage current. In this way, the threshold voltage of the device can be better controlled by varying the dielectric layer thickness [19]. 

While these methods were the first to be proposed to fabricate normally-off GaN HEMTs, they present some limitations, essentially related to the control of reproducibility (at a nanometric level) of the plasma etch process, V_th_ instabilities with increasing the operation temperature, etc. [20]. 

Today, the most popular solutions for normally-off GaN HEMTs are the “cascode” configuration, the “p-GaN gate” and the “recessed gate hybrid MISHEMT”. These technologies will be described in more detail in the following sections, discussing their advantages and limitations.

### 3.1. Cascode Configuration

**Figure 2:**
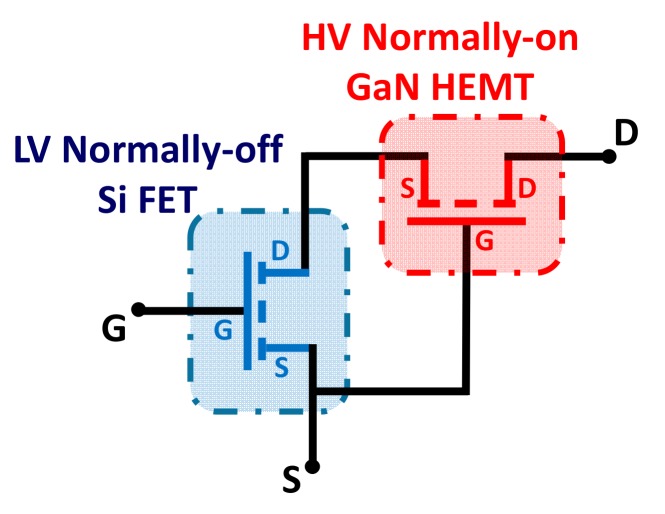
Normally-off GaN transistor obtained with the “cascode” configuration, combining a high-voltage (600 V) normally-on GaN high electron mobility transistors (HEMT) with a low-voltage (30 V) normally-off Si MOSFET.

A normally-off GaN HEMT can be realized adopting the so called “cascode” configuration, which has been previously used both in Si and 4H-SiC transistors to obtain a stable threshold voltage [21]. The “cascode” circuit, schematically shown in Figure 2, consists in the series connection of a low-voltage normally-off Si metal oxide semiconductor field effect transistor (MOSFET) with a high-voltage normally-on GaN HEMT. The two devices are connected in such a way that the output (drain–source) voltage of the MOSFET determines the input (gate–source) voltage of the HEMT. The devices share the same channel current in the on-state, while the blocking voltage is distributed between them in the off-state. 

The “cascode” working principle can be summarized as follows. When the Si MOSFET is turned on with a positive gate bias above its threshold voltage, the normally-on GaN HEMT gate voltage is close to zero and the device is turned on. Hence, for any applied voltage to the drain terminal, the current will flow through the normally-on GaN HEMT and the Si MOSFET, as they are connected in series [21]. On the other hand, when the Si MOSFET is turned off by removing the gate voltage, an applied bias to the drain terminal will create a negative voltage between the gate and source electrodes of the GaN HEMT. Consequently, the 2DEG channel will be pinched off, and any further increase of the drain voltage will be supported by the GaN HEMT [21].

With the “cascode” configuration, a positive and stable threshold voltage (V_th_ > 0) is obtained like in a Si MOSFET. At the same time, the system maintains the benefits provided by GaN materials, i.e., a low series resistance of the 2DEG in the on-state and a high electric field strength in the off-state.

Since some years, normally-off GaN HEMTs using the “cascode” configuration rated at 600 V are commercially available [22]. However, while the “cascode” configuration has the advantage that can be driven by the conventional MOSFET drivers, this solution exhibits some drawbacks. As an example, the series connection of the two devices increases the package complexity, and introduces parasitic inductances that affect the switching performance of the cascode configuration. Moreover, the high temperature operation is limited by the presence of a Si device. 

It is also important to point out that the “cascode” configuration work well when the GaN HEMT has a relatively high on-resistance with respect to the Si MOSFET (typically a 30 V device). In fact, since the on-resistance increases with increasing the rated breakdown voltage, the “cascode” approach is advantageous when the normally-on GaN HEMT is high-voltage and the Si MOSFET is low-voltage [23]. As an example, a 600 V GaN HEMT “cascode” will have only 3% added on-resistance due to the Si MOSFET. On the other hand, for lower targeted breakdown, the on-resistance of the GaN HEMT decreases and, hence, the contribution of the Si MOSFET becomes significant. Hence, the “cascode” approach is practically advantageous for applications above 200 V [23]. 

For the aforementioned reasons, today the power electronics community is continuously pushing towards the development of “real” normally-off GaN HEMT solutions, instead adopting the “cascode” configuration.

### 3.2. p-GaN Gate

**Figure 3:**
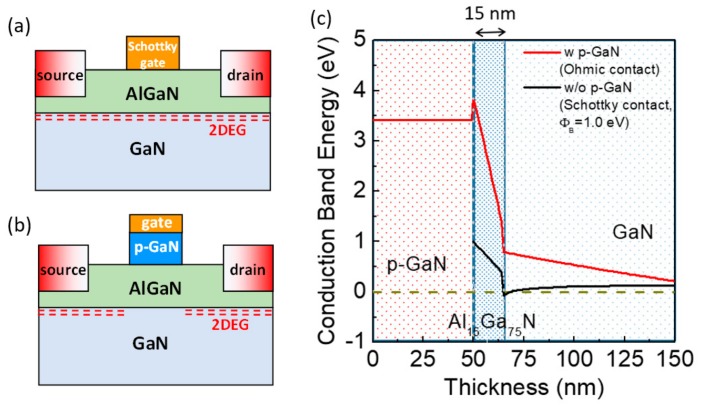
Schematic of a normally-on HEMT with a Schottky gate (**a**) and normally-off HEMT with a p-GaN gate (**b**); conduction band diagrams of a p-GaN/AlGaN/GaN heterostructure simulated for a p-GaN layer (3 × 10^19^ cm^−3^) of 50 nm, an AlGaN thickness of 15 nm and Al concentration of 15% (**c**). The Fermi level (dashed line) lies below the conduction band, which indicates the depletion of the two-dimensional electron gas (2DEG) and a normally-off condition. For comparison, the band diagram of an AlGaN/GaN heterostructure with a conventional Schottky gate is reported (normally-on).

The structure of a normally-off HEMT with a p-GaN gate is schematically depicted in Figure 3a, together with an example of band structure of a p-GaN/AlGaN/GaN system (Figure 3b). In particular, with respect to a standard normally-on HEMT with a Schottky gate, the presence of a p-GaN cap layer raises the AlGaN conduction band above the Fermi level. 

The band structure reported in Figure 3b was obtained by a commercial Poisson solver, for a p-GaN layer of 50 nm (with a doping level of 3 × 10^19^ cm^−3^), an AlGaN thickness of 15 nm and Al concentration of 15%. Under these conditions, the 2DEG can be depleted below the gate electrode and the normally-off operation is obtained, i.e., a positive gate bias will be needed to restore the electron charge in the channel.

Historically, the normally-off GaN HEMT with a p-AlGaN gate was first proposed by Uemoto et al. [24], and it exhibited a threshold voltage V_th_ = 1.0 V and a breakdown V_B_ = 800 V. 

In order to achieve an efficient depletion of the 2DEG and V_th_ > 0 the properties of the AlGaN/GaN heterostructure (i.e., thickness of the AlGaN barrier and Al-concentration) must be appropriately defined [25,26,27,28]. Typically, in a normally-off p-GaN/AlGaN/GaN heterostructure the AlGaN barrier layer thickness is in the range of 10–15 nm, while the Al concentration is in the order of 15–20%. Moreover, a high doping level of the p-GaN layer (> 10^18^ cm^−3^) is typically required for an efficient depletion of the region at the metal-gate/p-GaN interface [29]. In this sense, one of the key elements to improve the threshold voltage V_th_ for a fixed Mg-concentration of the p-GaN layer is to increase the Mg electrical activation. This latter can be done by appropriate p-GaN layer growth parameters and annealing conditions [30]. However, it is known that at temperatures above 500 °C in in the presence of hydrogen in the atmosphere, Mg-H complexes can form, either passivating the acceptors or compensating them by creation of donor states, leading to a reduced hole concentration [31]. Hence, device manufacturers should also take care that the Mg active concentration is not decreased during device processing (e.g., annealing for Ohmic contacts).

Another relevant aspect in normally-off HEMT technology with the p-GaN gate is the choice of the metal gate. In fact, the threshold voltage of the device V_th_ is related, among other things, to the metal/p-GaN Schottky barrier height. Several papers in the last years discussed on the influence of metal gate work-function on the electrical behavior of p-GaN gate HEMTs [29,32,33,34,35]. 

In the early studies, a Pd-based Ohmic gate was adopted on the p-AlGaN cap layer, which allowed to improve the hole injection and the current capability of the device [24]. For that reason, the device was also named “gate injection transistor” (GIT) [24]. However, in that work the fabrication process and the properties of this Pd/p-AlGaN system were not disclosed. 

On the other hand, more recently, TCAD simulations of the metal/p-GaN/AlGaN/GaN system predicted [32,33] that a Schottky metal gate on p-GaN should give a higher V_th_ and lower leakage with respect to an Ohmic gate. Meneghini et al. [33] showed that a WSiN-based Schottky gate, instead of a standard Ni/Au Ohmic contact to p-GaN, is able to increase the transistor gate voltage swing and reduces the gate leakage current by about four orders of magnitude in the on-state. In general, a high gate leakage in GaN HEMTs translates into a continuous power consumption and heating of the gate driver. Hence, a good Schottky barrier in p-GaN gate HEMTs ensures the absence of large current injection at the gate side, enabling a lower power consumption. For that reason, the use of a Schottky gate on p-GaN is today preferred to the Ohmic gate solution.

Table 2 reports some literature data on normally-off p-GaN gate HEMTs. As can be seen, several metals have been used as Schottky gate contact to p-GaN (Mo-, Ni-, Ti-based stacks, etc.). Presently, a good solution is represented by the use of a TiN gate, owing to its thermal and chemical stability and the processing compatibility [30,36,37,38]. TiN metal gates are typically defined by a stacked-gate self-aligned patterning approach, etching the TiN metal gate and the p-GaN in one sequence. Posthuma et al. [30] showed that an optimization of the Mg activation in the p-GaN layer can result in a positive V_th_ shift in p-GaN HEMTs with a TiN gate, up to V_th_ = 2.1 V.

From the data reported in Table 2, a dependence of the threshold voltage V_th_ on the metal work function cannot be easily deduced. In fact, the experimental behavior of metal/p-GaN Schottky barriers is typically influenced from surface preparation and/or post-annealing conditions. 

In this context, the fabrication flow chart of normally-off HEMT with a p-GaN gate is the most critical part is the definition of the p-GaN gate, as the p-GaN layer must be removed selectively from the access regions and left in under the gate. In a “self-aligned” process, the metal gate is deposited first (“gate first”) and used at the same time as metal contact for the p-GaN and as hard mask for the dry etch of p-GaN in the access regions. While this approach can simplify the fabrication procedure, the high temperature required for the source-drain Ohmic contacts (>800 °C) [39] can promote thermal reactions between metal gate and p-GaN degrading the electrical quality of the barrier.

**Figure 4:**
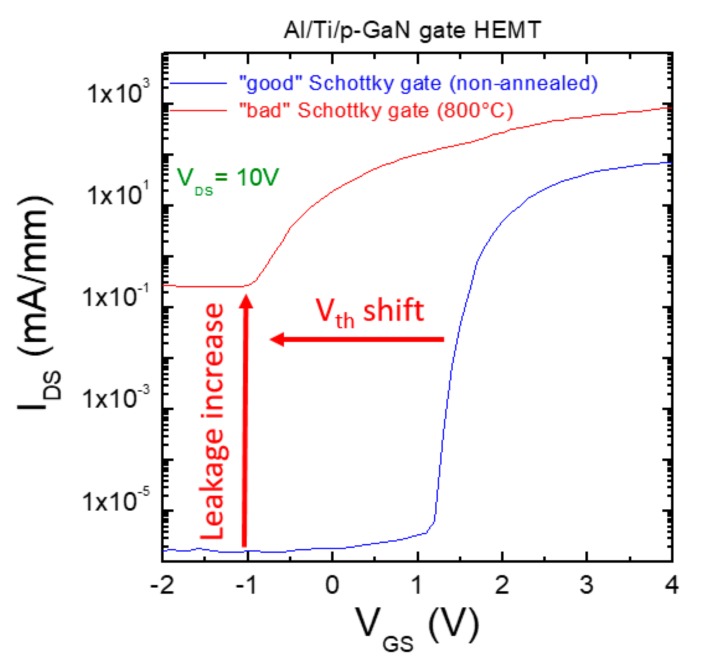
Transfer characteristics (I_DS_ vs. V_GS_) at V_DS_ = 10 V of normally-off p-GaN gate HEMTs fabricated with a “good” Al/Ti/p-GaN Schottky contact (non-annealed) and with a “bad” Al/Ti/p-GaN Schottky contact (annealed at 800 °C).

Greco et al. [34] demonstrated that a normally-off operation with V_th_ = 1.5 V can be obtained with a Ti/Al metal gate. However, the structural changes at the Al/Ti/p-GaN interface upon thermal annealing (400–800 °C) leads to a decrease of the Schottky barrier height, which in turn results into an increase of the leakage current and a negative shift of V_th_ [34]. This effect is visible in Figure 4, reporting the transfer characteristics of the p-GaN HEMTs with a good Al/Ti/p-GaN Schottky gate (non-annealed) and a bad Al/Ti/p-GaN Schottky gate (annealed at 800 °C) [34]. 

To avoid the inconvenience related to the leakage of the gate, the GaN device community is oriented to adopt fabrication processes employing low thermal budgets or stable metallizations to p-GaN compatible with a “gate first” flow. As an example, TiN is often used for these purposes, and employed in commercial devices [23].

Recently, Lükens et al. [40] presented a “self-aligned” process for a normally-off HEMT with p-GaN gate, stable at high temperatures. In particular, they employed a “gate first” process using a Mo-based layer acting simultaneously as gate metallization and p-GaN etch hard mask [40]. Interestingly, the Mo gate was able to sustain the annealing for source-drain Ohmic contacts at 825 °C without degradation of the barrier.

In this context, it is worth reminding that the great interest towards GaN-based heterostructures grown on large area Si substrates is given by the possibility of power device fabrication inside the existing Si CMOS lines. Hence, common to all the aforementioned approaches is the need of Au-free metallizations, to guarantee the full compatibility of the p-GaN gate technology with the Si CMOS lines requirements [39].

Today, although the p-GaN gate is the only “real” normally-off HEMT solution that has already reached the commercialization, there are still some reliability issues that are object of intensive investigations. As an example, normally-off p-GaN HEMTs are often affected by gate leakage current, when stressing the gate at forward voltage above 5–6 V. In fact, in forward bias the p-GaN layer acts as a depletion layer for the Schottky contact. The device is equivalent to a back-to-back diode, i.e., a metal/p-GaN Schottky diode in series with a p-GaN/AlGaN/GaN p-n junction [45]. Under high negative bias, the p-n diode blocks the gate current, while at high positive gate bias the Schottky gate junction blocks the current. When a high stress voltage is applied to the gate electrode, a large voltage drop and an electric field occur in the depletion region of the p-GaN close to the metal interface, thus promoting the formation percolation paths in the p-GaN layer that are responsible for the leakage current [45,46]. In this sense, the thermal and electrical stability of the metal/p-GaN interface is an important requirement. Moreover, the roughness of the p-GaN gate sidewalls (defined by dry etch process) can be also one of the causes of leakage current in these devices [47]. An overview on the reliability issues in normally-off HEMT with p-GaN gate is given in reference [35].

For sake of completeness, it must be mentioned that other processes to achieve normally-off HEMTs with p-type GaN-based cap layers have been objects of investigation in the last years. 

In some works, the aim was to eliminate in the fabrication flow chart the critical processing step of the selective p-GaN dry etch. As an example, the selective area growth of the p-GaN layer in the gate region can be a promising approach to avoid the need of the selective dry etch of the p-GaN layer in the access region [48]. While this technology eliminates the issues related to the plasma-induced damage in the access regions, controlling the electrical properties and the uniformity of the selectively grown p-GaN layer is a challenging aspect. Alternatively, another interesting approach that has been proposed to avoid the plasma etching of the p-GaN layer consists in the “deactivation” of Mg-dopant in the p-GaN layer in the access regions by means of a hydrogen plasma treatment [44,49]. In fact, hydrogen atoms are able to passivate the Mg-acceptors in the p-GaN by the formation of Mg-H complexes [31]. 

On the other hand, Mizutani et al. [50] successfully fabricated normally-off AlGaN/GaN HEMTs using a p-In_0.23_Ga_0.77_N cap layer, instead of p-GaN. The basic idea of this approach was to take advantage of the polarization-induced field in the InGaN cap and negative charge in the p-InGaN cap. In fact, in a p-InGaN/AlGaN/GaN structure, the conduction band at the AlGaN/GaN interface is strongly raised, thus leading to normally-off operation. Using this approach, a threshold voltage V_th_ = 1.2 V was obtained [50].

More details on these processes can be found in a recent review paper on normally-off GaN HEMTs with p-GaN gate [28]. 

In spite of the critical processing of the gate region, the normally-off HEMT with a p-GaN gate represents still the only commercially available solution. 

### 3.3. Recessed Gate Hybrid MISHEMT

**Figure 5:**
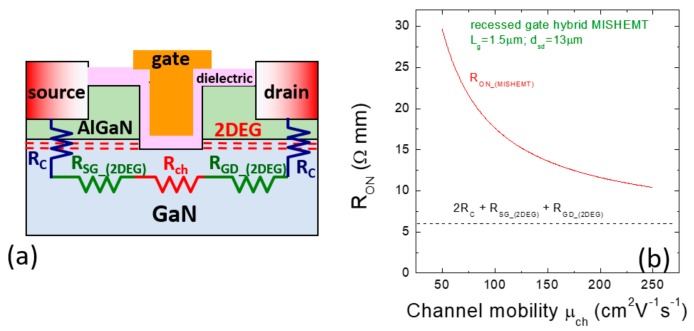
(**a**) Schematic of a recessed gate hybrid MISHEMT. The contributions to the total series resistance are also indicated. (**b**) Total resistance of the MISHEMT *R_ON_(MISHEMT)_* as a function of the channel mobility calculated for a device with L_g_ = 1.5 μm and d_sd_ = 13 μm.

A schematic of the recessed gate hybrid GaN MISHEMT is reported in Figure 5a. In this device, in the gate region the AlGaN barrier layer is removed by plasma etch and the recessed GaN region is passivated by an insulator. This device is a hybrid transistor connecting in series the recessed MIS-channel with two access regions having a low resistance (thanks to the presence of the 2DEG). Zi et al. [51] and Ikeda et al. [52] were among the first who simulated and demonstrated at R&D level recessed gate hybrid GaN MISHEMTs with threshold voltage V_th_ up to +2 V, specific on-resistance R_ON_ < 10 mΩcm^2^ and breakdown in the kV range, proposing this solution for power switching applications.

As can be seen in Figure 5a, the total on-resistance of this transistor *R_ON_(MISHEMT)_* is given by the sum of different contributions:

*RO_N_(MISHEMT)_* = 2*R_C_* + *R_SG_(2DEG)_* + *R_GD_(2DEG)_* + *R_ch_*, (1)

where *R_C_* is the contact resistance of the source/drain electrodes, *R_SG_(2DEG)_* and *R_GD_(2DEG)_* are the access resistance contributions and *R_ch_* is the resistance of the channel region, i.e., where the 2DEG has been removed. The channel resistance *R_ch_* is proportional to the gate length *L_g_* and decreases with increasing the channel mobility *μ_ch_* [51].

Obviously, the recessed gate region is the most important part of this normally-off transistor. In particular, the surface roughness of the recessed area, the presence of electrically active defects, and the electronic quality of the insulator/GaN interface can have a significant impact on the channel mobility *μ_ch_* and, hence, on the total on-resistance *R_ON_(MISHEMT)_* of the device.

For that reason, the insulator/GaN interfaces in the recessed channel of MISHEMTs are continuously object of investigations. 

Figure 5b reports the on-resistance *R_ON_(MISHEMT)_* as a function of the channel mobility *μ_ch_* calculated for a recessed gate hybrid GaN MISHEMT, assuming a source-drain distance d_sd_ = 13 μm [53] and a gate length L_g_ = 1.5 μm. In the calculation, realistic values of the contact resistance (*R_C_* = 0.5 Ω.mm) and semiconductor sheet resistance (*R_SH_* = 400 Ω/sq) were considered. The resistive contribution of recessed channel was assumed considering our experimental measurements on a recessed SiO_2_/GaN MISHEMTs, which gave a channel sheet resistance of 7.3 kΩ/sq for a channel mobility value of 110 cm^−2^·V^−1^·s^−1^ [54].

As can be seen in Figure 5b, for the selected device layout the series resistance decreases of a factor of three with increasing the channel mobility from 50 to 200 cm^2^·V^−1^·s^−1^. When the carrier mobility in the recessed channel region is increased, the series resistance *R_ON_(MISHEMT)_* tends to saturate, because the resistive contribution of the recessed channel *R_ch_* becomes less important with respect to the other contributions (*R_c_*, *R_SG_(2DEG)_* and *R_GD_(2DEG)_*).

Clearly, in a recessed hybrid MISHEMT controlling the properties of the insulator/GaN interface (i.e., by reducing the roughness, the interface states density, etc.) is mandatory to optimize the channel mobility and, hence, to minimize the device series resistance.

The parameter, which is typically used to describe the electrical behavior of the channel in a recessed gate hybrid MISHEMT, is the so-called “field effect mobility” *μ_FE_*, defined as:

(2) $$\mu_{FE}=\frac{L_{g}}{WC_{ox}V_{DS}}\left(\frac{\partial I_{DS}}{\partial V_{GS}}\right)$$

where *C_ox_* is the gate insulator capacitance per unit area, *L_g_* and *W* are the channel length and width, respectively.

Typically, the curves of the field effect mobility *μ_FE_* as a function of the gate bias *V_GS_* increase up to a maximum *μ_FE(max)_* and then slightly decrease at high *V_GS_*.

**Figure 6:**
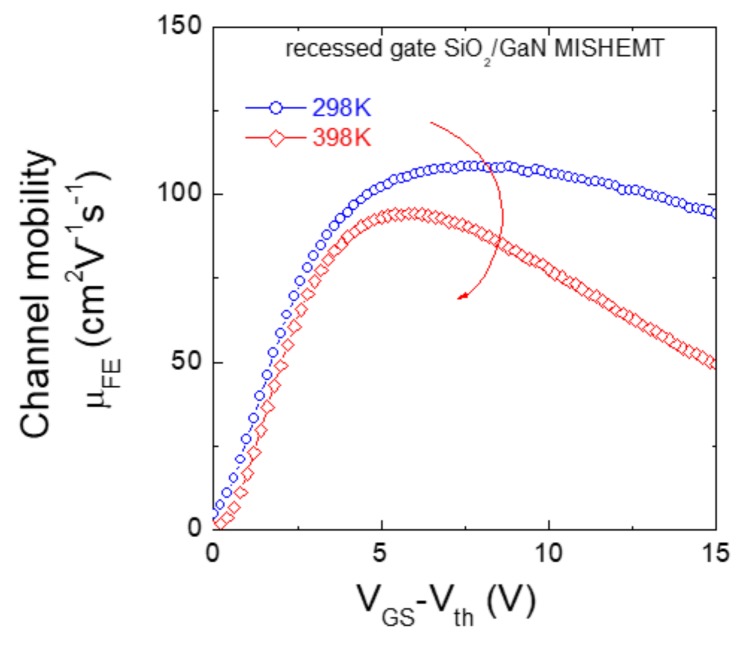
Field effect channel mobility μ_FE_ as a function of V_GS_–V_th_ of a recessed gate SiO_2_/GaN MISHEMT, measured at room temperature (298 K) and at 398 K.

Fiorenza et al. [54] recently analyzed the behavior of the field effect mobility in recessed gate hybrid MISHEMTs employing SiO_2_ as gate insulator, with respect to several parameters (temperature, surface roughness, electric field, dielectric quality, etc.). In particular, this work highlighted the importance of the reduction of the interface state density in the insulator/GaN system to increase the field effect mobility and reduce the specific on-resistance [54]. As an example, Figure 6 shows the field effect mobility of the recessed gate SiO_2_/GaN MISHEMT measured at 298 K and 398 K. As can be seen, with increasing the temperature a slight decrease of the maximum peak mobility occurs, which is accompanied by a variation of the slope of the mobility curves at high V_GS_–V_th_. This behavior was explained considering different scattering contributions in the total mobility. The results indicated that Coulomb and phonon scattering are the dominant mechanisms limiting the channel mobility of the recessed gate MISHEMT.

Table 3 reports a survey of literature data on relevant parameters for normally-off recessed gate hybrid MISHEMTs employing different gate insulators.

In particular, the values of the peak mobility *μ_FE(max)_* of these devices vary in the range 38–251 cm^2^·V^−1^·s^−1^, while the threshold voltage V_th_ is usually in the range +1 to +2 V. For devices with a typical gate length of 1.5 μm, the values of the on-resistance *R_ON_* in the operation conditions (i.e., at sufficiently high V_GS_) are in the range 7.2–26 Ω.mm [57,62,63,64], as can be also seen in the calculation shown in Figure 5b. 

Clearly, in recessed gate hybrid MISHEMTs the preparation of the channel surface and post annealing processes are very important. In terms of device manufacturability in Si CMOS lines, a full recess etch of the AlGaN barrier is preferred, since it is a robust process with a large process window, which limits the V_th_ dispersion caused by non-uniform residual AlGaN on the wafer. Moreover, also the deposition process of the dielectric need to be accurately controlled, as it can lead to a degradation of the channel region, especially when this deposition is carried out at high temperatures. The data in Table 3 refer to devices with different geometries and different processes to prepare the recessed channel. Hence, a direct comparison of the electrical parameters of these devices is not straightforward. However, as a general trend, the highest values of the peak mobility (*μ_FE(max)_* > 150 cm^2^·V^−1^·s^−1^) correspond to the lowest values of on-resistance (*R_ON_* < 10 Ω·mm).

As can be seen in Table 3, several dielectric materials have been adopted nowadays for recessed gate normally-off MISHEMTs, the most common being Al_2_O_3_, SiO_2_ and SiN_x_.

Al_2_O_3_ is typically deposited by Atomic Layer Deposition (ALD) at moderate temperatures (<300 °C). On the other hand, high quality SiN_x_ films can be deposited by low-pressure chemical vapor deposition (LPCVD), but high deposition temperatures (800 °C) maybe required to reduce the defect density and improve the film quality. 

To overcome this limitation, Cai et al. [59] recently proposed a "channel-engineering" method based on an oxygen-plasma treatment followed by in-situ annealing before SiN_x_ gate dielectric deposition on GaN. The formation of a crystalline GaON nanophase with a high thermal stability and low interface states density was the key factor for the optimization of the performance of SiN_x_/GaN normally-off MISHEMT, in terms of mobility, threshold voltage and on-resistance [59].

Currently, in spite of many promising results reported on GaN-based recessed gate hybrid MISHEMTs, this technology still suffers from threshold voltage instability issues, related to charge trapping effects inside the gate insulator [66,68,69,70,71]. 

**Figure 7:**
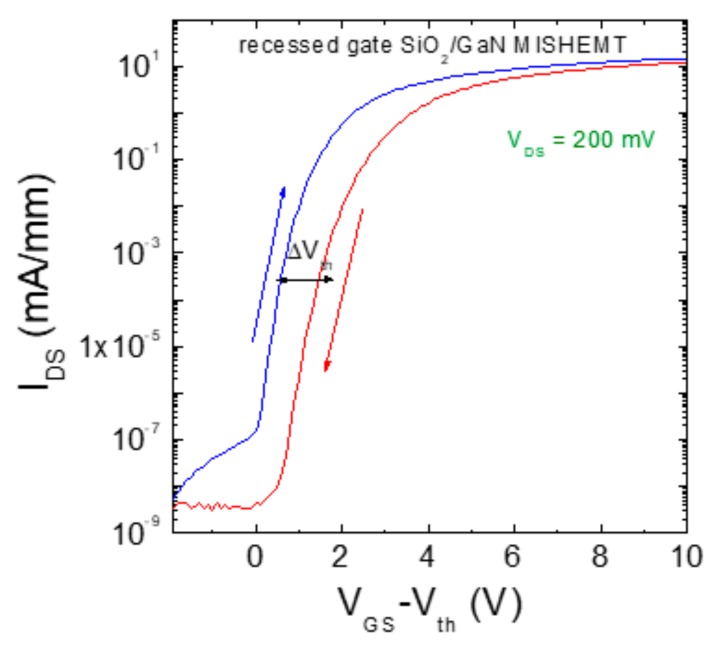
Transfer characteristics (I_DS_ vs V_G_-V_th_) acquired at V_DS_ = 200 mV in a recessed gate MISHEMT under forward and reverse bias sweep, showing a shift of the threshold voltage (ΔV_th_).

As an example, Figure 7 shows the transfer characteristics (I_DS_ vs V_GS_-V_th_) of a recessed gate SiO_2_/GaN MISHEMT acquired under a forward and backward bias sweep. As can been, after the backward bias sweep the transfer characteristics are positively shifted with respect to the forward sweep. The positive threshold voltage shift (ΔV_th_) indicates a negative charge trapping in the gate insulator. 

Typically, a threshold voltage instability in MISHEMTs can produce two effects. In the case of a positive *V_th_* shift, like that observed in Figure 7, a degradation of the on-resistance of the device occurs, due to the larger bias needed to obtain the same current value. On the other hand, in the case of a negative *V_th_* shift (positive charge trapping), the normally-off behavior can be lost.

Clearly, the occurrence of these charge trapping effects at the gate dielectric represents a big issue for recessed gate MISHEMTs. Hence, this approach has not yet reached an adequate maturity to be commercialized and still remains object of R&D investigations of the GaN community. 

## 4. Summary and Outlook

This paper gave a brief overview on the state-of-the-art of normally-off technology for GaN HEMTs. This topic is very important, as GaN transistors are expected to penetrate significantly the power semiconductor device market in the next years. The presence of the 2DEG in GaN-based heterostructure makes HEMT devices normally-on by nature. Hence, developing reliable solutions for normally-off HEMTs is still a challenge for GaN technology.

As pointed out in the preceding sections, although it is possible to use normally-on HEMTs in power electronics applications by adopting the “cascade” configuration, today the market demands "real" normally-off transistors. 

Among the different approaches that have been proposed to achieve normally-off operation in GaN HEMTs, the most important ones are the p-GaN gate and the recessed gate hybrid MISHEMT. In this context, it is very important to understand the benefits and drawbacks of these technologies.

A direct comparison of the behavior of normally-off GaN HEMTs with p-GaN gate with recessed gate MISHEMTs has been reported by Marcon et al. [72]. In their work, they compared two devices with identical geometries (L_g_ = 1.5 μm, d_sd_ = 7.25 μm and W = 100 μm) fabricated on AlGaN/GaN heterostructures with the same buffer technology.

**Figure 8:**
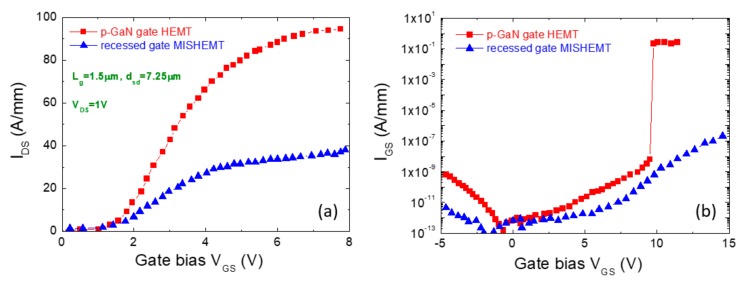
(**a**) Comparison of the transfer characteristics (at V_DS_ = 1 V) of normally-off HEMT with p-GaN gate and recessed gate MISHEMT. (**b**) Forward leakage current of the same devices. The figure has been adapted from reference [72] with the permission of the authors.

Figure 8a shows the direct comparison of the transfer characteristics for the two normally-off transistors. As can be seen, while both devices exhibit a similar V_th_ of about 1.5 V, a larger output current is carried by the p-GaN gate HEMT with respect to the recessed gate MISHEMT. Such a difference is due to the larger series resistance of the recessed gate MISHEMT below the gate region with respect to the p-GaN gate. In fact, as also discussed in Section 3 (Equation (2)), the channel contribution to the on-resistance in a recessed gate MISHEMT linearly scales with the gate length L_g_ [51]. Hence, in recessed gate hybrid MISHEMTs the gate length is a very important design parameter, and values of L_g_ < 1.5 μm are typically required to have good performances. 

On the other hand, the recessed gate MISHEMT exhibits a lower forward leakage current with respect to the p-GaN gate, as can be seen in Figure 8b. This latter is due to the presence of the gate dielectric, which suppresses the leakage current and increases the breakdown (>9 V).

Another interesting term of comparison is the hysteresis of the devices. In particular, while this phenomenon is practically absent in p-GaN gate HEMTs, it is still a limitation in recessed gate hybrid MISHEMTs, due to the presence of interface states and bulk trap in the gate dielectric. Hence, further work on the gate dielectric is required to optimize the technology of normally-off recessed gate MISHEMTs.

The advantages and disadvantages, in terms of processing and performances, of the three main normally-off GaN HEMT designs described in the previous sections are summarized in Table 4.

In conclusion, today, the p-GaN gate approach remains the only one that is commercially available. However, significant R&D progress has been achieved also in the recessed gate MISHEMT technology and continuous efforts are devoted to improve further this technology. 

Clearly, both approaches still present several open issues that must be addressed to optimize the reliability and manufacturability. Moreover, each solution exhibits advantages and disadvantages, in terms of processing and performances in different applications. Hence, it can be expected that the choice of the normally-off design will depend on the targeted application. In this context, the p-GaN gate will be probably more suitable for the low- and medium-voltage applications, while the MISHEMT technology will be used for applications at higher voltages. Moreover, the market penetration of normally-off GaN-based solutions in power electronics will be related also to the decrease of the materials cost and to the improvement of the material quality, which in turn is affects the device reliability. In this scenario, from an academic point of view, great efforts of the research community will be required in the next years to reach a more complete understanding of the physics of GaN-based materials and devices.

## Figures and Tables

**Table 1:** Relevant physical and electronic properties of gallium nitride (GaN) compared with silicon (Si) and silicon carbide (4H-SiC). The figures of merit (FOM) for power and high frequency electronics, normalized with respect to Si, are also reported. The table was compiled taking the data from Studies [7,8] and the references therein.

Property	Si	4H-SiC	GaN
Bandgap (eV)	1.12	3.2	3.4
Critical field E_cr_ (MV/cm)	0.25	3	4
Dielectric constant ε	11.8	9.7	9.5
Saturation velocity v_s_ (10^7^ cm/s)	1	2	3
Electron mobility µ (cm^2^/Vs)	1350	800	1300* (* 2DEG)
Intrinsic carrier concentration n_i_ (cm^−3^) at 300 K	10^10^	10^−7^	10^−10^
Thermal conductivity k (W/cmK)	1.5	4.9	1.3
**Figure of merit (FOM)**	–	–	–
JFOM (v_s_E_cr_)^2^/(v_s_E_cr_)^2^Si	1	576	2304
BFOM (εμE_cr_^3^)/(εμE_cr_^3^)Si	1	842	3175
BHFOM (μE_cr_^2^)/(μE_cr_^2^)Si	1	85	246

**Table 2:** Survey of literature data on normally-off HEMTs with p-GaN gates obtained using different Schottky contacts.

Metal Gate	p-GaN Thickness t and Doping N_A_	Threshold Voltage V_th_ (V)	Ref.
Mo _(100nm)_/Ni _(20nm)_	t = 80 nm, N_A_ = 3 × 10^19^ cm^−3^	1.08	[40]
Mo/Ti/Au	t = 60 nm, N_A_ ~ 2 × 10^18^ cm^−3^	1.9	[33]
Ni	t = 100 nm, N_A_ = 2 × 10^19^ cm^−3^	1.23	[32,41]
Ni/Au	t = 60 nm, N_A_ ~ 2 × 10^18^ cm^−3^	1.8	[33]
Ni _(25nm)_/Au _(120nm)_	t = 60 nm, N_A_ = 1 × 10^18^ cm^−3^	1.7–2.1	[33]
Ni _(20nm)_/Au _(200nm)_	t = 50 nm, N_A_ = 3 × 10^18^ cm^−3^	0.48	[42]
Pd _(50nm)_/Au _(150nm)_	t = 70 nm, N_A_ not given	1.0	[43]
Ti _(50nm)_/Au _(150nm)_	t = 70 nm, N_A_ not given	1.2	[44]
Ti/Au	t = 60 nm, N_A_ ~ 2 × 10^18^ cm^−3^	1.7	[33]
Ti _(30nm)_/Al _(170 nm)_	t = 50 nm, N_A_ = 3 × 10^19^ cm^−3^	1.5	[34]
TiN	t = 70 nm, N_A_ = 1 × 10^18^ cm^−3^	1.6	[36]
TiN	t = 60 nm, N_A_ not given	2.1	[30]
W	t = 100 nm, N_A_ = 1 × 10^19^ cm^−3^	3.0	[32,41]
WSiN	N.A.	1.87	[35]

**Table 3:** Survey of literature data on normally-off recessed gate hybrid GaN MISHEMTs, employing different gate insulators.

Gate Insulator	Insulator Processing	µ_FE (Max_) (cm^2^·V^−1^·s^−1^)	V_th_ (V)	R_ON_(MISHEMT)_ (Ω mm)	Ref.
SiO_2 (60nm)_	PECVD on SAG of 5 nm GaN	166	3.7	N.A. (FATFET)	[55]
SiO_2 (60nm)_	PECVD	94	2.4	N.A. (FATFET)	[55]
SiO_2 (50nm)_	PECVD + Post Annealing at 850 °C in N_2_	110	0.7	N.A. (FATFET)	[54]
SiN _(20nm)_	PECVD	120	5.2	22 Ω.mm at V_GS_ = 13 V	[56]
SiN _(2nm) LT_/SiN _(15nm) HT_	LT-PECVD at 300 °C + HT-LPCVD at 780 °C	160	2.37	13.2 Ω.mm at V_GS_ = 15 V	[57]
SiN _(17nm) HT_	HT-LPCVD at 780 °C	38	1.28	20 Ω.mm at V_GS_ = 15 V	[57]
SiN _(20nm)_	LPCVD on photo-electrochemical recess	203	1.2	12.2 Ω.mm at V_GS_ = 17 V	[58]
SiN_(15nm)_	SiN LPCVD at 780 °C	49	0.8	26 Ω.mm at V_GS_ = 15 V	[59]
GaON/SiN_(15nm)_	ICP in O_2_ + SiN LPCVD at 780°C	141	1.3	12 Ω.mm at V_GS_ = 15 V	[59]
Al_2_O_3 (30nm)_	ALD + Post Annealing at 800 °C in N_2_	225	2	7.8 Ω.mm at V_GS_ = 6 V	[60]
Al_2_O_3 (38nm)_	TMAH treated GaN surface	55	3.5	27 Ω.mm at V_GS_ = 15 V	[61]
Al_2_O_3 (10nm)_	ALD + Post Annealing at 400 °C in N_2_	251	1.7	9.8 Ω.mm at V_GS_ = 8 V	[62]
Al_2_O_3 (20nm)_	ALD + Post Annealing at 400 °C in N_2_	148	2.9	7.2 Ω.mm at V_GS_ = 9 V	[63]
Al_2_O_3 (30nm)_	ALD + SAG of access regions	170	3.5	9.5 Ω.mm at V_GS_ = 12 V	[64]
Al_2_O_3 (18nm)_	ALD + Post Annealing at 400 °C in N_2_	65	7.6	19.5 Ω.mm at V_GS_ = 14 V	[65]
AlN _(7nm)_/SiN _(7nm )_	MOCVD	180	1.2	N.A. (FATFET)	[66]
Al_2_O_3 (5nm)_/SiN _(25nm)_	SiN LPCVD at 780 °C	122	1.7	12.9 Ω.mm at V_GS_ = 18 V	[67]

**Table 4:** Advantages and disadvantages of the three main normally-off GaN HEMT solutions in terms of processing and performances: “cascode” configuration, p-GaN gate and recessed gate hybrid MISHEMT.

Normally-Off Design	Advantages	Disadvantages
Cascode	-Use of standard MOSFET gate driver -Stable V_th_ > 0 of the Si MOSFET	-Package complexity -Optimization of Si MOSFFET needed in each application -Not suitable for low voltage (<600 V) and high frequency (>1 MHz) applications
p-GaN gate	-Low resistance under the gate -No dielectric issues	-Optimization of p-GaN etching needed for low access resistance and better reliability -Limited positive gate voltage swing
Recessed gate hybrid MISHEMT	-Large forward breakdown -Standard device driving in applications	- Not suitable for low voltage application (100 V), due to the gate channel resistance -Critical impact of the gate module (interface and dielectric) on device performances (R_ON_ and reliability)
